# Effect of temperature variation on the corneal endothelial cell during femtosecond laser-assisted cataract surgery compared with conventional phacoemulsification cataract surgery: A prospective study

**DOI:** 10.1097/MD.0000000000049632

**Published:** 2026-07-31

**Authors:** Shanshan Gu, Yu Cao, Jian Wu, Min Ji, Yemeng Huang, Qin Hu, Dongyun Sun, Huaijin Guan

**Affiliations:** a Eye Institute, Affiliated Hospital of Nantong University, Nantong, China.

**Keywords:** anterior chamber, endothelial cell loss, FLACS, temperature

## Abstract

To evaluate whether the corneal endothelium experiences increased temperatures and thermal damage during femtosecond laser-assisted cataract surgery (FLACS), we performed comparative in vivo studies. These studies compared FLACS with conventional phacoemulsification cataract surgery (CPS) systems. In this study, 410 eyes with grade II or III nuclear sclerotic cataracts undergoing either CPS (n = 213) or FLACS (n = 197) were examined. All eyes received balanced salt solution at 21°C or 29°C during phacoemulsification. In FLACS, a SoftFit patient interface (PI) was used at 21°C or 29°C. The surface temperature and intraocular temperature were recorded using a thermometer. Postoperative therapies were identical across all groups. Endothelial cell loss (ECL) was measured with a specular microscope before and 1 week post-surgery. The average temperature in the anterior chamber was similar after FLACS and CPS. FLACS used less cumulative dissipated energy (CDE) and resulted in lower ECL than CPS. However, no significant difference was found in mean CDE or ECL between subcohorts with different PI temperatures. The same applied to subcohorts with different balanced salt solution temperatures. At a PI temperature of 21°C, both anterior chamber temperature and ECL were lower in the first 10 minutes after femtosecond laser (FL) treatment than afterward. FL does not cause temperature increases in the anterior chamber. In contrast, FLACS reduces CDE and ECL compared with CPS. Additionally, lowering PI temperature and performing FL before phacoemulsification – especially during the first 10 minutes – may help protect the corneal endothelium.

## 1. Introduction

Cataracts are the leading cause of blindness and visual impairment worldwide. The contribution of cataracts to blindness worldwide is likely to increase due to the aging population. Therefore, it has become important to establish the safest and most effective surgical techniques for cataract surgery.^[1]^ Conventional phacoemulsification cataract surgery (CPS) was initiated in 1967 by Charles Kelman.^[2]^ This procedure uses high-intensity ultrasound (US) energy to fragment and emulsify the lens. The lens is disintegrated inside the capsular bag and aspirated through the phacoemulsification (phaco) probe. The probe consists of a hollow needle made of titanium alloy. This method has several advantages, such as a small incision, minimal tissue damage, quick repair, small postoperative astigmatism, and fast vision recovery. This method has been demonstrated to be an effective and tolerable treatment for cataracts.^[3]^ At the same time, complications are rare but can be serious and endanger the patient’s sight. Corneal endothelial damage still represents one of the most common and serious complications.^[4]^

In humans, the corneal endothelium is a vulnerable monolayer of hexagonal cells with limited regenerative capacity. Because corneal endothelial cells lack the ability to regenerate, the loss of these cells is compensated only by their migration, enlargement, and increased heterogeneity.^[5]^ The corneal endothelium acts as a barrier and an active fluid pump system, dehydrating the corneal stroma by its pumping action, which maintains the transparency and regular thickness of the cornea.^[6]^ Corneal endothelial damage impairs its cell density, structure, and function, leading to corneal edema. When the damage is excessive, it results in irreversible bullous keratopathy.^[4]^ The mean endothelial cell density (ECD) considered normal for adults is approximately 2500 cells/mm^2^, with corneal edema and decompensation occurring when it falls below 500 cells/mm^2^.^[7]^ A recent national survey^[8]^ of bullous keratopathy in Japan reported that cataract surgery was the most common cause of penetrating keratoplasty (24.2%). Although the safety of CPS has been dramatically improved, the prevention of corneal endothelial damage during phaco is still an important concern for cataract surgeons. With increasing expectations of fast visual recovery after cataract surgery, corneal cell integrity and functionality are important. Endothelial cell loss (ECL) after uncomplicated cataract surgery has been reported to vary from 4 to 25%.^[9]^ The reasons for endothelial damage during phaco are not yet completely understood. ECL can be influenced by several pre- and intraoperative factors. Preoperative factors that influence ECL include older age, small pupil diameter, a harder nucleus, and shorter axial length.^[10]^ Damaging factors associated with surgery, such as the collision of lens nuclear fragments with the corneal endothelium, are well known.^[11]^ Other intraoperative factors include the size and design of the incision,^[12]^ the ophthalmic viscoelastic device used,^[13]^ the volume of balanced salt solution (BSS) used,^[14]^ excessive duration of US oscillation,^[15]^ collapsing cavitation bubbles,^[16]^ or irrigating pressure.^[17]^ Furthermore, the temperature increase caused by US energy in the anterior chamber should be considered another important harmful factor leading to postoperative cell loss. Research has shown that thermal energy generated during cataract surgery, identified as the main heat source, can lead to mechanical trauma.

The physiological temperature in the anterior chamber of the human eye, on average, is 30 ± 1.4°C.^[18]^ Furthermore, the endothelium is vulnerable to thermal damage.^[19]^ The maximum temperature that can be tolerated is 32°C.^[20]^ The higher the temperature in the anterior chamber, the more severe the damage to the corneal endothelial cells. Self-heating of the probe and friction have been identified as heat sources, causing a temperature increase around the phacoemulsifier tip.^[21]^ The damage induced by the thermal energy around the tip during surgery has been a concern. Occlusion of the tip tends to cause rapid temperature increases and an increased risk of corneal burns. The corneal endothelium, in particular, is at risk because of its proximity to the tip of the vibrating probe of the phacoemulsifier.^[22]^ In China, due to the influence of traditional concepts, elderly people used to undergo surgery late. Therefore, the lens nucleus is larger and harder, the US energy used in the operation is higher, and the operation time is longer. The resulting thermal effects are likely to burn the corneal endothelial cells.

Femtosecond laser (FL) technology was introduced in cataract surgery (femtosecond laser-assisted cataract surgery, FLACS) in 2009.^[23]^ Early steps of cataract surgery, including laser capsulotomy, lens fragmentation, astigmatic corneal relaxing incisions, and clear corneal incisions, are performed using FLACS. Several studies suggest that FLACS offers numerous advantages, including a precisely executed circular capsulotomy, reduced phaco time, less collateral tissue damage, a lower risk of intraocular lens shift, and an improved safety profile. It also significantly reduces the amount of US energy required for lens nucleus work-up during the operation. Given that US energy is directly related to ECL, FLACS causes less damage to corneal endothelial cells and may benefit eyes with low preoperative endothelial cell counts.^[24–26]^ Numerous comparisons of FLACS and CPS have been published as the number of cases studied continues to increase.^[17,26–28]^ However, the effect of FLACS and CPS on the temperature of the anterior segment of the eye has not been well evaluated. In the present study, we aimed to determine whether temperature increases occur in the anterior chamber during FLACS, to explore the procedure’s thermal safety, and to assess its effects on the corneal endothelium compared with CPS.

## 2. Information and methodology

**Figure 1.**
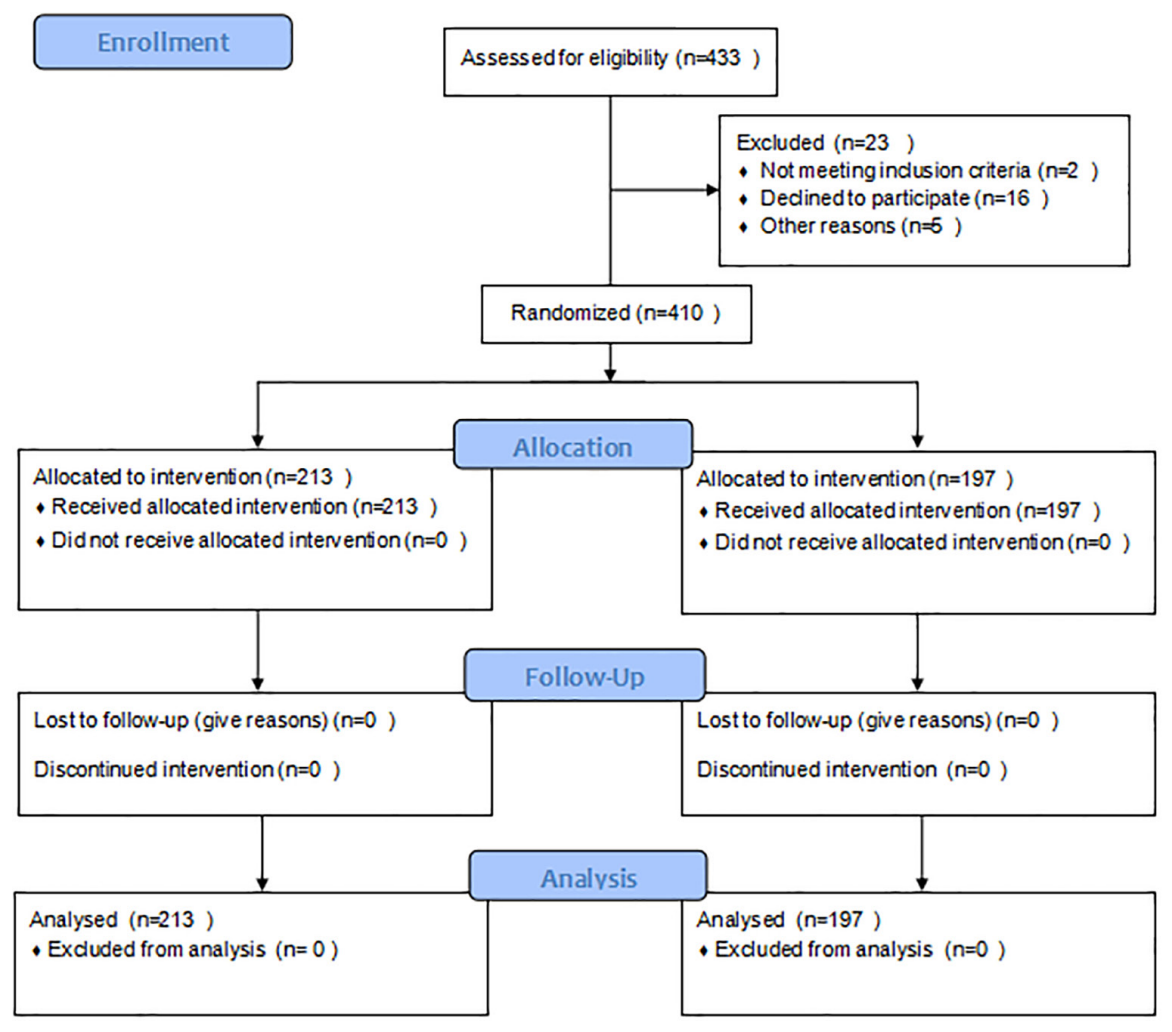
Patient enrollment and study flow diagram.

This prospective, non-randomized, comparative study included patients scheduled for cataract extraction. Patients were allocated to either the FLACS or CPS group based on patient preference after detailed counseling on the benefits and costs of each procedure. The sample size was calculated a priori using G*Power software. Based on a pilot study, we assumed a mean difference in ECL of 5% between groups with a standard deviation of 10%. To achieve 90% power with a 2-sided α of 0.05, a minimum of 171 eyes per group was required. Accounting for a potential 10% dropout rate, we aimed to enroll at least 188 eyes in each group. The procedures were performed using FLACS (197 eyes) or CPS (203 eyes) at the Department of Ophthalmology, the Affiliated Hospital of Nantong University from March to October 2024 (the patient enrollment and study flow diagram is shown in Fig. 1). The baseline characteristics, including gender and cataract grade, were comparable between the 2 groups. The following inclusion criteria were exercised: patients older than 18 years with visually serious cataracts and the ability to sign informed consent forms, with pupils dilating more than 7 mm. Moreover, the recruited patients had nuclear opacity classified as grade II or III nuclear sclerosis (NS) based on the Emery–Little classification.^[29]^ The exclusion criteria were the presence of a history of corneal or intraocular surgery, severe dry eye, corneal scars, corneal dystrophy, history of herpes keratitis, signs of keratoconus, history of uveitis, pseudoexfoliation syndrome, glaucoma, traumatic cataract, low preoperative ECD (<1500 cells/mm^2^), lack of cooperation, and ocular tremor. Furthermore, all intraoperative complications, such as capsular rupture and zonular dehiscence, were also excluded. We ensured that the differences between the groups were attributable to treatment factors (surgical methods, temperature of SoftFit patient interface [PI], temperature of BSS), but not other factors.

All patients underwent a complete preoperative examination. This included history taking, uncorrected and corrected distance visual acuity tests, slit-lamp biomicroscopy, applanation tonometry, and dilated fundus evaluation. Moreover, all patients were NS graded by an extensively experienced surgeon (Jian Wu). Only those patients whose lenses were assessed to be NS grade II or III were included in this study. Next, 3 photographs of each cornea were taken, and the corneal ECD was assessed using a noncontact specular microscope (EM-3000; TOMEY). The method is fully automated. The mean of the readings was calculated and marked as the final reading. All patients who were enrolled received the same treatment regimen consisting of a combination of an antibiotic and nonsteroidal anti-inflammatorydrop 4 times daily initiated 3 days prior to surgery.

The PI, which consisted of a curved interface and contact lens, was used for corneal applanation (Alcon Laboratories, Fort Worth, Tex.). The interface surface had a diameter of 10.8 mm and a curvature of 8.3 mm. The operating room temperature was set to 21°C. We measured the average corneal surface temperature of patients upon entering the room, which was 29°C. Therefore, the PIs were placed in an incubator (MIR-H163-PC; Panasonic) at either 21°C or 29°C for at least 1 day before surgery. Moreover, we used BSS (Alcon) as the irrigating solution, either at 21°C or heated to 29°C throughout the surgeries. The BSS was stored in the operating room or incubator at 30°C for at least 1 day prior to surgery. Then, we used the fluid warmer (F8000, FLIGHT, China) to maintain constant temperature of the BSS. After flowing from the tube, the temperature of the BSS that flowed from the phaco tip we measured was 21°C or 29°C. On the day of surgery, every patient underwent dilatation of the pupil by instillation of 1 drop of tropicamide phenylephrine eye drops (Santen) every 15 minutes for 45 minutes. Surgeries were performed under the cover of topical anesthesia using proparacaine hydrochloride eye drops (Alcon).

Cataract surgeries were performed using either CPS or FLACS. Phacoemulsification and irrigation/aspiration were conducted with the Centurion Vision System (Alcon), equipped with an intrepid balanced ultrasonic tip featuring a small sleeve for microincisions. The phaco-chop technique was applied as previously reported.^[30]^ FLs were performed using the LenSx laser machine (Alcon) as previously reported. After hydro dissection, lens removal was performed using a Centurion machine with a phaco-chop technique. The aspheric intraocular lens model 251 (HOYA) was implanted in all eyes. Intraoperative complications, such as anterior capsule tears, posterior capsule tears, and posterior capsule ruptures, were recorded. All patients were operated by the same experienced cataract surgeon (Huaijin Guan).

Postoperatively, data from the Centurion phacoemulsification machine were manually recorded and entered into an Excel spreadsheet for each patient. These data included total US time, cumulative dissipated energy (CDE), and a detailed fluidics profile. The Centurion Vision System uses CDE as a value for phaco energy. This is calculated as (phaco time × average phaco power%) + (torsional time × average torsional amplitude × 0.4) (The factor 0.4 represents approximate reduction in heat dissipated at the incision compared to conventional phaco).^[31]^ All parameter settings during phacoemulsification or irrigation/aspiration were the same.

**Figure 2.**
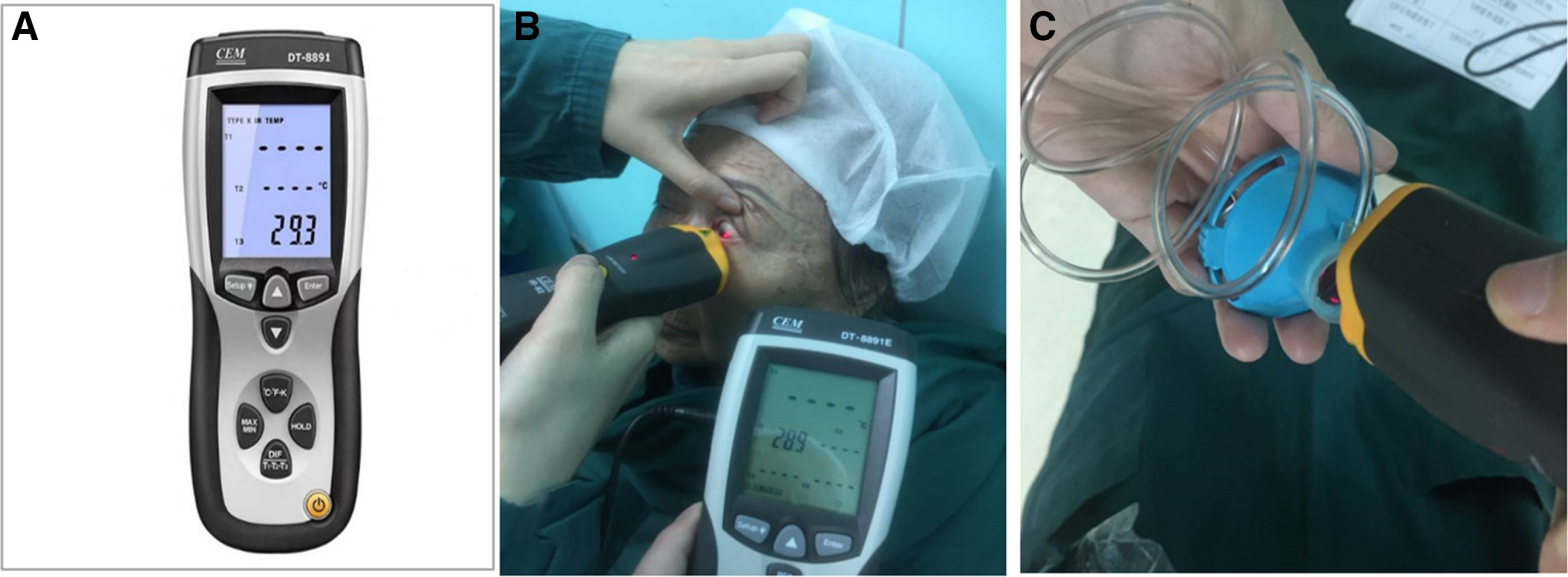
Temperature was measured by the thermometer (A). The surface temperatures of the cornea and PI were obtained by using a noncontact infrared thermometer (B, C). PI = patient interface.

**Figure 3.**
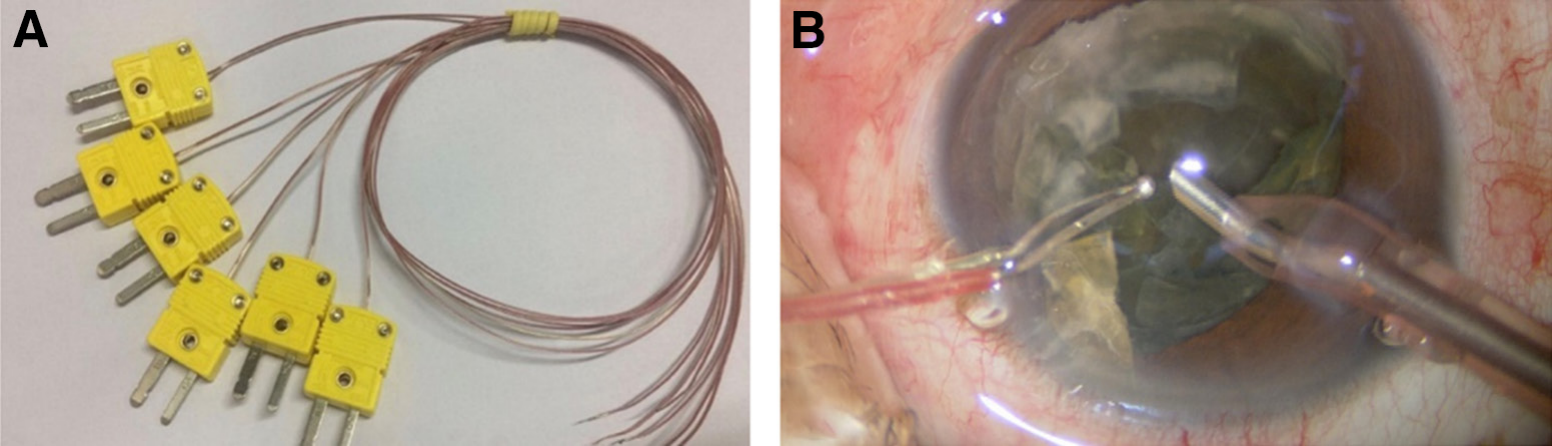
Temperature in the eye was measured by type K thermocouples (A). Insertion of the thermocouple in the eye from the side-port at 3:00 direction (B).

Temperatures were measured by the thermometer DT-8891E (CEM, Shenzhen, China) (Fig. 2A). The surface temperatures of the cornea and PI were obtained using a noncontact infrared thermometer (IR-82; CEM) (Fig. 2B, C). The temperatures inside the anterior chamber before phaco were measured invasively by inserting type K thermocouples (TT-K-30-SLE; SMT; USA) (Fig. 3A) in the anterior chambers through the main corneal incisions, situated 5 mm from the limbus in the longitudinal direction. The temperatures in the capsular bags during phaco were measured using type K thermocouples inserted from the side-port in the 3:00 direction, 2.5 to 3 mm from the tips of the phacoemulsification instrument (Fig. 3B). Temperatures were constantly recorded during phacoemulsification, with readings taken every 1 second. The ophthalmologist who performed the postoperative endothelial cell count was masked to the group assignment and the temperature conditions of the surgery.

The corneal ECD was assessed 1 week postoperatively as before. The percentage of ECL was calculated using the following formula, where pre = preoperative and post = postoperative:


$$ECL\left(\;\%\;\right)=\frac{ECD_{pre}-ECD_{post}}{ECD_{pre}}$$

All descriptive statistical analysis was performed using SPSS software (version 23.0; SPSS, Inc.). Data were presented as numbers and percentages for nominal variables and as mean and standard deviation for continuous parameters. The characteristics of the patients in 2 groups were compared using an independent t-test, whereas 1-way analysis of variance (ANOVA) and pairwise comparison were used to analyze the between groups in 3 groups. For multiple pairwise comparisons, a Bonferonni correction was applied to adjust the significance level. A *P*-value of <.05 was considered statistically significant.

## 3. Results

Originally, 410 patients met the inclusion and exclusion criteria and were included in the study. We comprised 213 eyes that underwent cataract surgery using CPS and 197 eyes that underwent cataract surgery using FLACS. All of the enrolled patients completed the 1-week follow-up. Table 1 shows the demographic data of the study population. Preoperatively, there was no statistically significant difference in gender or cataract density between the 2 groups. Tables 2–5 show the preoperative and postoperative clinical parameters.

**Table 1:** Patient characteristics of the whole cohort, FLACS and CPS subcohorts.

Characteristics	Whole cohort (n = 410)	FLACS (n = 197)	CPS (n = 213)	*P*-value
Age (a)	66.21 ± 11.92	65.91 ± 13.18	66.58 ± 10.20	.85
Gender, n (%)				.74
M	170 (41.5)	80 (40.61)	90 (42.25)	
F	240 (58.5)	117 (59.39)	123 (57.75)	
NS grade, n (%)				.51
II	277 (67.6)	130 (65.99)	147 (69.01)	
III	133 (32.4)	67 (34.01)	66 (30.99)	

CPS = conventional phacoemulsification cataract surgery; F = female; FLACS = femtosecond laser-assisted cataract surgery; M = male.

**Table 2:** Preoperative and postoperative various values for FLACS and CPS.

	FLACS	CPS	
	21°C PI, 21°C BSS	21°C BSS	29°C BSS
Eyes (n)	61	132	81
Preop T on corneal surface	28.97 ± 0.87	28.95 ± 1.55	29.02 ± 1.58
T in the anterior chamber before phaco	29.62 ± 1.34*	30.38 ± 1.13	30.23 ± 0.82‡
T in the lens capsule during phaco	21.33 ± 1.33	21.05 ± 1.01†	28.14 ± 3.28‡
CDE (U/S)	4.89 ± 2.65*	6.62 ± 4.50	6.59 ± 4.86‡
% ECD loss	9.17 ± 5.01*	16.56 ± 11.75	16.27 ± 12.59‡

BSS = balanced salt solution, CDE = cumulative dissipated energy, CPS = conventional phacoemulsification cataract surgery, ECD = endothelial cell density, FLACS = femtosecond laser-assisted cataract surgery.

* Comparison between FLACS and CPS under 21°C BSS, *P* < .05.

† Comparison between CPS under 21°C and 29°C BSS, *P* < .05.

‡ Comparison between FLACS under 21°C BSS and CPS under 29°C BSS, *P* < .05.

**Table 3:** Preoperative and postoperative various values for FLACS.

	21°C PI, 21°C BSS	29°C PI, 21°C BSS	29°C PI, 29°C BSS
Eyes (n)	61	76	60
Preop T on corneal surface	28.97 ± 0.87	28.99 ± 1.16	29.04 ± 1.17
T in the anterior chamber before phaco	29.62 ± 1.34*	30.02 ± 1.16	30.05 ± 0.99‡
T in the lens capsule during phaco	21.33 ± 1.33	21.36 ± 0.99†	28.10 ± 1.82‡
CDE (U/S)	4.89 ± 2.65	5.00 ± 3.44	5.01 ± 2.57
% ECD loss	9.17 ± 5.01	10.16 ± 6.63	9.75 ± 4.18

BSS = balanced salt solution, CDE = cumulative dissipated energy, ECD = endothelial cell density, FLACS = femtosecond laser-assisted cataract surgery.

* Comparison between FLACS with 21°C and 29°C PI, *P* < .05.

† Comparison between FLACS under 21°C BSS and CPS under 29°C BSS, *P* < .05.

‡ Comparison between FLACS with 21°C PI, 21°C BSS and 29°C PI, 29°C BSS, *P* < .05.

**Table 4:** Preoperative and postoperative various values for FLACS and CPS under 29°C BSS.

	FLACS with 29°C PI		CPS	
	II	III	II	III
Eyes (n)	40	21	54	27
Preop T on corneal surface	29.06 ± 1.01	28.99 ± 1.46	28.99 ± 1.54	29.08 ± 1.68
T in the anterior chamber before phaco	30.10 ± 1.05	29.97 ± 0.88	30.24 ± 0.86	30.20 ± 0.74
T in the lens capsule during phaco	28.09 ± 1.90	28.11 ± 1.69	28.18 ± 0.91	28.05 ± 1.13
CDE (U/S)	3.81 ± 1.49*	7.41 ± 2.60	5.39 ± 4.75‡	9.00 ± 4.21§
% ECD loss	8.21 ± 2.82*	12.81 ± 4.82†	14.15 ± 13.40‡	20.50 ± 1.69§

BSS = balanced salt solution, CDE = cumulative dissipated energy, CPS = conventional phacoemulsification cataract surgery, ECD = endothelial cell density, FLACS = femtosecond laser-assisted cataract surgery.

* Comparison of FLACS between NS grade II and III, *P* < .05.

† Comparison of NS grade III between FLACS and CPS, *P* < .05.

‡ Comparison of NS grade II between FLACS and CPS, *P* < .05.

§ Comparison of CPS between NS grade II and III, *P* < .05.

**Table 5:** Preoperative and postoperative various values for FLACS under 21°C BSS.

	21°C PI		29°C PI	
	<10 min	>10 min	<10 min	>10 min
Eyes (n)	21	40	27	49
Preop T on corneal surface	28.79 ± 0.99	29.07 ± 0.80	29.01 ± 1.08	28.97 ± 1.21
T in the anterior chamber before phaco	28.92 ± 0.99*	29.98 ± 1.36†	29.77 ± 0.95‡	30.33 ± 1.24
T in the lens capsule during phaco	21.25 ± 1.23	21.37 ± 1.39	21.37 ± 1.05	21.36 ± 0.96
CDE (U/S)	4.57 ± 1.94	5.06 ± 2.96	4.92 ± 2.71	5.04 ± 3.80
% ECD loss	7.29 ± 2.86*	10.15 ± 5.62	9.36 ± 4.08	10.60 ± 7.69

BSS = balanced salt solution, CDE = cumulative dissipated energy, ECD = endothelial cell density, FLACS = femtosecond laser-assisted cataract surgery.

* Comparison between in and over 10 min after FL with 21°C PI, *P* < 0.05.

† Comparison of over 10 min after FL with 21°C and 29°C PI, *P* < .05.

‡ Comparison of in 10 min after FL with 21°C and 29°C PI, *P* < .05.

Table 2 shows the mean temperature in the anterior chamber was lower after FL than in the CPS group when PI was 21°C. As the temperature of BSS increased, the temperature in the capsular bag during phaco increased. In terms of surgical outcomes, an independent samples *t* test revealed a statistically significant difference in the mean CDE and ECL between the CPS and FLACS groups. The FLACS subcohort required a lower mean CDE and had a lower mean ECL. At the same time, there were no differences in the mean CDE and ECL between the CPS subcohorts with different BSS temperatures. Tables S1 and S2, Supplemental Digital Content 1 and 4 show that when we further classified the patients according to the NS, we obtained the same outcome as before. In addition, even less CDE was required, and less ECL was obtained in NS grade II than in III.

Next, we compared the parameters of placing the PI at 21°C or 29°C and increasing the BSS temperature to 29°C. We found that as the temperature of PI increased, the temperature in the anterior chamber increased. As the temperature of BSS increased, the temperature in the lens capsule during phaco increased. There was no difference in the mean CDE and ECL between the subcohorts with different temperatures of PI or BSS (Table 3). Tables S3 and S4, Supplemental Digital Content 2 and 5 shows that when we further classified the patients according to the NS, we found no difference in temperature in the anterior chamber between the subcohorts with 21°C PI or 29°C PI. We obtained the same outcome as before when we raised the temperature of BSS. In addition, less CDE was required, and less ECL was obtained in the NS grade II than in the NS grade III.

Table 4 shows that there was no statistically significant difference in the mean temperature in the anterior chamber between the CPS and FLACS when PI increased to 29°C. In addition, less CDE was required and less ECL was obtained in NS grade II subcohorts than in NS grade III. Moreover, the FLACS subcohort required a lower mean CDE and obtained a lower mean ECL, except for comparing the CDE of NS grade III.

We maintained a constant temperature of the BSS at 21°C, classified the data for further analysis, and found that the mean temperature in the anterior chamber when PI at 21°C was lower than that at 29°C. Moreover, the temperature in the anterior chamber ten minutes after FL was lower than over ten minutes when PI was 21°C. In addition, the ECL in ten minutes after FL was lower than over ten minutes when PI was 21°C (Table 5). Furthermore, we found no statistically significant difference in the relationship between the mean temperature in the anterior chamber and CDE, ECL in ten minutes and over ten minutes after FL when PI increased to 29°C (Table S5, Supplemental Digital Content 3).

## 4. Discussion

Currently available FLs use neodymium: glass light with a wavelength of 1053 nm, delivering short pulses (10–15 nanoseconds) of energy at near-infrared wavelength, which is not absorbed by thin or almost transparent tissue. The laser light can be focused on the targeted area within the anterior segment with a 3 μm spot size and an accuracy of ±5 μm, without directly affecting the penetrated tissues.^[32]^ The laser can perform corneal incisions, anterior capsulotomy, and fragmentation of the lens nucleus. However, FL has always been considered a cold technology.^[33]^ One study measured the highest temperature rise during lens fragmentation by theFL Victus, ranging from 3.53°C to 5.13°C. Additionally, the temperature rise cannot be harmful to human corneas.^[34]^ An in vitro analysis^[35]^ was carried out that showed negligible anterior chamber temperature changes during precision pulse capsulotomy. Such minute temperature increases are safe and unlikely to cause endothelial cell or collateral tissue trauma. However, in our study, we found that FL does not induce a temperature increase in the anterior chamber, which is estimated to be because FL does not generate heat itself or because FL acts on the lens and does not affect the temperature outside the capsular bag. In addition, we found that the temperature in the anterior chamber after FL was lower when PI was 21°C.

One study^[36]^ measured the temperatures of the rabbit lens after exposure to sunlight. The results provided evidence that an increase in ambient temperature can raise the temperature of the lens. Another study^[37]^ stated that as heat enters the eye through the corneal surface, the temperature of the aqueous humor (AH) next to the cornea is raised first. The anterior and posterior chambers were filled with AH that was constantly in motion due to thermally induced buoyancy. The study also revealed that convective heat flux contributed to more than 95% of the total heat flow inside the anterior chamber due to AH flow. One study^[38]^ has shown that the greater the temperature difference between the corneal and anterior chambers, the faster the AH circulation. At the same time, the average heat flux across the cornea increases with the temperature differences. In our study, we found that PI affects the temperature in the anterior chamber. When the temperature of the PI increased to 29°C, the temperature of the anterior chamber was higher than at 21°C. However, the temperature increase of the PI has no effect on the temperature inside the capsular bag. It is estimated that the action of BSS makes the influence of AH on the temperature inside the capsular bag negligible.

An in vitro analysis^[4]^ measured the temperature of the AH during phaco in animal eyes. The data clearly showed that increases in US power caused an increase in the anterior chamber temperature, and increased temperature in the AH is one of the most common causes of corneal endothelial dysfunction. Another^[39]^ reported a maximum temperature of 34.1°C in the AH and a postoperative ECL of 7%. Another study found an ECL of up to 10% if the aqueous temperature was increased from 29.1°C to 33.4°C. However, Buschschluter et al^[40]^ demonstrated that there was no relationship between the temperature increase inside the anterior chamber during common phaco and endothelial cell damage. In addition, 1 study^[41]^ showed that hypothermic solution reduces the energy in the process of the operation with less damage to corneal endothelial cells, avoids complications and sequelae, and improves the efficacy of cataract surgeries, with it adopted in phaco for hard nucleus cataracts. However, Praveen et al^[42]^ found that cooled BSS Plus at 10°C did not affect postoperative corneal parameters and concluded that the use of moderately cooled BSS Plus had no detectable effect and benefit on the outcome of phaco. The other study^[6]^ found that the solution temperature with room temperature or refrigerated fortified BSS did not influence ECL.

Our observations suggested that when the BSS temperature was increased to 29°C, the temperature inside the capsular bag increased, but it had no effect on ECL. However, we raised the temperature only within the low end of the average physiological temperature range. The continuous flow of BSS or ophthalmic viscoelastic device (iviz; Bausch & Lomb) likely protects the endothelium against heat. Therefore, the maximum temperature during the operation does not exceed the upper limit of the physiological temperature range. Currently, we have no research on reducing the temperature or refrigerating the BSS. We speculate that colder BSS would further lower the temperature in the capsular bag and reduce ECL when PI is set at a lower temperature. We will further reduce the temperature of the BSS in the next study. The optimal hypothermia temperature for intraocular irrigation in cataract surgery needs to be verified in future studies. At the same time, we will further raise the temperature of BSS in animal experiments to study the effect of warming on endothelial cells.

Several published studies^[25,26]^ have raised concerns that FLACS may reduce the amount of required US energy used in cataract surgery, a factor known to be directly related to ECL. It is believed that FLACS, compared with CPS, might result in reduced ECL. One study^[17]^ showed that FLACS resulted in a decrease in CDE and less ECL when compared to standard phaco at 1 month postoperatively. Weiner et al^[43]^ found significantly lower effective phaco time as well as a reduction in ECL postoperatively in the FL group. In our study, we also found a statistically significant decrease in CDE during FLACS when compared to standard phaco and showed less ECL postoperatively. The differences were less pronounced in a study by Krarup et al,^[27]^ with ECL at 3 months postoperatively of 11.4% (after laser treatment) and of 13.9% following CPS. Another study^[44]^ also found no statistically significant difference in ECL at 6 months between 30 eyes that received FLACS (for capsulotomy, lens fragmentation, and corneal incision using the LenSx Laser System) and 30 eyes that underwent standard phaco.

This study showed that FLACS resulted in decreased CDE and lower ECL compared with CPS. Moreover, when PI was at 21°C, the anterior chamber temperature was lower during the first ten minutes after FL than after ten minutes. Moreover, the temperature of the anterior chamber within ten minutes was lower when PI was at 21°C compared to 29°C. The lower temperature likely mitigates the effects of hyperthermia during phaco on the cornea, and the ECL was also lower. After ten minutes, it was estimated that the temperature increased due to AH flow.

There are a few limitations to this study. First, this was a non-randomized study. Patients self-selected into FLACS or CPS groups after counseling, which introduced potential selection bias. Although we applied strict exclusion criteria, such as a history of corneal disease and low preoperative ECD, we did not collect data on other factors, including diabetes, axial length, corneal thickness, and baseline endothelial morphology. We minimized confounding by matching baseline characteristics between groups. Thus, observed differences likely resulted from treatment factors. Second, the invasive placement of thermocouples may have altered AH dynamics and temperature, potentially affecting study outcomes. Third, the follow-up period was limited. This study had a short-term follow-up period, as many participants were unable to complete subsequent follow-ups regularly. Therefore, future studies with longer follow-up periods and expanded sample sizes are needed to determine the effects of these techniques on the corneal endothelium.

## 5. Conclusions

In summary, no temperature increase was observed in the anterior chamber during FL. Increasing the BSS temperature up to 29°C does not cause damage to the cornea. FLACS resulted in a decrease in CDE and lower ECL compared with standard phaco. We also demonstrated that reducing the PI temperature and performing FL followed by phaco may protect the corneal endothelium, especially during the first 10 minutes. Further studies are required to validate these findings. Therefore, the use of FLACS, particularly with a cooled PI and performed prior to phacoemulsification, represents a potentially safer option for preserving corneal endothelial cells, especially in cases with hard nuclei.

## Author contributions

**Conceptualization:** Jian Wu, Qin Hu, Dongyun Sun, Huaijin Guan.

**Data curation:** Shanshan Gu, Min Ji, Yemeng Huang, Dongyun Sun, Huaijin Guan.

**Formal analysis:** Shanshan Gu.

**Funding acquisition:** Huaijin Guan.

**Investigation:** Shanshan Gu, Yu Cao, Min Ji.

**Methodology:** Yu Cao, Qin Hu, Huaijin Guan.

**Project administration:** Jian Wu, Yemeng Huang.

**Resources:** Huaijin Guan.

**Software:** Yu Cao, Min Ji, Qin Hu.

**Validation:** Jian Wu, Dongyun Sun.

**Visualization:** Qin Hu, Dongyun Sun.

**Writing – original draft:** Shanshan Gu, Yu Cao, Yemeng Huang.

**Writing – review & editing:** Shanshan Gu, Huaijin Guan.










