# Supplementary material for: Effect of temperature variation on the corneal endothelial cell during femtosecond laser-assisted cataract surgery compared with conventional phacoemulsification cataract surgery: A prospective study
Source: Medicine (Baltimore). 2026 Jul 31;105(31):e49632. doi: 10.1097/MD.0000000000049632 (PMC13433043; doi:10.1097/MD.0000000000049632)
Supplement: Supplementary file 1 [file medi-105-e49632-s001.docx]

Table S1. Preoperative and postoperative various values for FLACS and CPS under 21°C BSS

|  | FLACS with 21°C PI | | CPS | |
| --- | --- | --- | --- | --- |
|  | II | III | II | III |
| Eyes (n) | 40 | 21 | 93 | 39 |
| Preop T of FL or phaco on corneal surface | 29.06±0.83 | 28..80±0.93 | 28.96±1.60 | 28.94±1.45 |
| T in the anterior chamber | 29.66±1.37 | 29.54±1.31^*^ | 30.38±1.05^&^ | 30.37±1.31 |
| T in the lens capsule during phaco | 21.24±1.55 | 21.50±0.75 | 21.31±1.02 | 21.42±1.00 |
| CDE (U/S) | 3.62±1.55^#^ | 7.31±2.64^*^ | 5.32±3.55^&^ | 9.70±5.04^^^ |
| % ECD loss | 6.41±1.92^#^ | 14.43±4.90^*^ | 14.35±10.12^&^ | 21.82±13.70^^^ |

#: Comparison of FLACS between NS grade II and III, *p*<0.05

^: Comparison of CPS between NS grade II and III, *p*<0.05

&: Comparison of NS grade II between FLACS and CPS, *p*<0.05

*: Comparison of NS grade III between FLACS and CPS, *p*<0.05
