# Supplementary material for: Effect of temperature variation on the corneal endothelial cell during femtosecond laser-assisted cataract surgery compared with conventional phacoemulsification cataract surgery: A prospective study
Source: Medicine (Baltimore). 2026 Jul 31;105(31):e49632. doi: 10.1097/MD.0000000000049632 (PMC13433043; doi:10.1097/MD.0000000000049632)
Supplement: Supplementary file 2 [file medi-105-e49632-s002.docx]

Table S3. Preoperative and postoperative various values for FLACS under 21°C BSS

|  | 21°C PI | | 29°C PI | |
| --- | --- | --- | --- | --- |
|  | II | III | II | III |
| Eyes (n) | 40 | 21 | 50 | 26 |
| Preop T of FL or phaco on corneal surface | 29.06±0.83 | 28.80±0.93 | 29.04±1.29 | 28.88±0.87 |
| T in the anterior chamber | 29.66±1.37 | 29.54±1.31 | 30.09±1.16 | 29.89±1.17 |
| T in the lens capsule during phaco | 21.24±1.55 | 21.50±0.75 | 21.27±1.04 | 21.54±0.86 |
| CDE (U/S) | 3.62±1.55^#^ | 7.31±2.64 | 3.52±2.43 | 7.84±3.32^^^ |
| % ECD loss | 6.41±1.92^#^ | 14.43±4.90 | 7.85±5.60 | 14.61±6.27^^^ |

#: Comparison of FLACS between NS grade II and III with 21°C PI, *p*<0.05

^: Comparison of FLACS between NS grade II and III with 29°C PI, *p*<0.05

&: Comparison of NS grade II between FLACS with 21°C and 29°C PI, *p*<0.05

*: Comparison of NS grade III between FLACS with 21°C and 29°C PI, *p*<0.05
