# Supplementary material for: Effect of temperature variation on the corneal endothelial cell during femtosecond laser-assisted cataract surgery compared with conventional phacoemulsification cataract surgery: A prospective study
Source: Medicine (Baltimore). 2026 Jul 31;105(31):e49632. doi: 10.1097/MD.0000000000049632 (PMC13433043; doi:10.1097/MD.0000000000049632)
Supplement: Supplementary file 3 [file medi-105-e49632-s003.docx]

Table S5. Preoperative and postoperative various values for FLACS with 29°C PI

|  | 21°C BSS | | 29°C BSS | |
| --- | --- | --- | --- | --- |
|  | < 10min | > 10min | < 10min | > 10min |
| Eyes (n) | 27 | 49 | 37 | 23 |
| Preop T of FL or phaco on corneal surface | 29.01±1.08 | 28.97±1.21 | 28.96±1.10 | 29.16±1.28 |
| T in the anterior chamber | 29.77±0.95 | 30.16±1.24 | 30.05±1.03 | 30.07±0.93 |
| T in the lens capsule during phaco | 21.37±1.05 | 21.36±0.96^*^ | 28.11±2.10^&^ | 28.08±1.28 |
| CDE (U/S) | 4.92±2.71 | 5.04±3.80 | 5.06±2.84 | 4.93±2.10 |
| % ECD loss | 9.36±4.08 | 10.60±7.69 | 9.66±4.72 | 9.89±3.24 |

#: Comparison between in and over ten minutes after FL under 21°C BSS, *p*<0.05

&: Comparison of in ten minutes after FL under 21°C and 29°C BSS, *p*<0.05

*: Comparison of over ten minutes after FL under 21°C and 29°C BSS, *p*<0.05
