# Supplementary material for: Effect of temperature variation on the corneal endothelial cell during femtosecond laser-assisted cataract surgery compared with conventional phacoemulsification cataract surgery: A prospective study
Source: Medicine (Baltimore). 2026 Jul 31;105(31):e49632. doi: 10.1097/MD.0000000000049632 (PMC13433043; doi:10.1097/MD.0000000000049632)
Supplement: Supplementary file 4 [file medi-105-e49632-s004.docx]

Table S2. Preoperative and postoperative various values for CPS

|  | 21°C BSS | | 29°C BSS | |
| --- | --- | --- | --- | --- |
|  | II | III | II | III |
| Eyes (n) | 93 | 39 | 54 | 27 |
| Preop T of phaco on corneal surface | 28.96±1.60 | 28.94±1.45 | 28.99±1.54 | 29.08±1.68 |
| T in the anterior chamber | 30.38±1.05 | 30.37±1.31 | 30.24±0.86 | 30.20±0.74 |
| T in the lens capsule during phaco | 21.31±1.02 | 21.42±1.00^*^ | 28.18±0.91^&^ | 28.05±1.13 |
| CDE (U/S) | 5.32±3.55^#^ | 9.70±5.04 | 5.39±4.75 | 9.00±4.21^^^ |
| % ECD loss | 14.35±10.12^#^ | 21.82±13.70 | 14.15±13.40 | 20.50±1.69^^^ |

#: Comparison of CPS between NS grade II and III under 21°C BSS, *p*<0.05

^: Comparison of CPS between NS grade II and III under 29°C BSS, *p*<0.05

&: Comparison of NS grade II between CPS under 21°C and 29°C BSS, *p*<0.05

*: Comparison of NS grade III between CPS under 21°C and 29C BSS, *p*<0.05
