# Supplementary material for: Effect of temperature variation on the corneal endothelial cell during femtosecond laser-assisted cataract surgery compared with conventional phacoemulsification cataract surgery: A prospective study
Source: Medicine (Baltimore). 2026 Jul 31;105(31):e49632. doi: 10.1097/MD.0000000000049632 (PMC13433043; doi:10.1097/MD.0000000000049632)
Supplement: Supplementary file 5 [file medi-105-e49632-s005.docx]

Table S4. Preoperative and postoperative various values for FLACS with 29°C PI

|  | 21°C BSS | | 29°C BSS | |
| --- | --- | --- | --- | --- |
|  | II | III | II | III |
| Eyes (n) | 50 | 26 | 40 | 20 |
| Preop T of FL or phaco on corneal surface | 29.04±1.29 | 28.88±0.87 | 29.06±1.01 | 28.99±1.46 |
| T in the anterior chamber | 30.09±1.16 | 29.89±1.17 | 30.10±1.05 | 29.97±0.88 |
| T in the lens capsule during phaco | 21.27±1.04 | 21.54±0.86^*^ | 28.09±1.90^&^ | 28.11±1.69 |
| CDE (U/S) | 3.52±2.43^#^ | 7.84±3.32 | 3.81±1.49 | 7.41±2.60^^^ |
| % ECD loss | 7.85±5.60^#^ | 14.61±6.27 | 8.21±2.82 | 12.81±4.82^^^ |

#: Comparison of FLACS between NS grade II and III under 21°C BSS, *p*<0.05

^: Comparison of FLACS between NS grade II and III under 29°C BSS, *p*<0.05

&: Comparison of NS grade II between FLACS under 21°C and 29°C BSS, *p*<0.05

*: Comparison of NS grade III between FLACS under 21°C and 29°C BSS, *p*<0.05
